# Supplementary material for: Dog Owners’ Perceptions of the Convenience and Value of Chewable Oclacitinib: Quantitative Survey Data from an International Survey
Source: Animals (Basel). 2024 Mar 19;14(6):952. doi: 10.3390/ani14060952 (PMC10967547; doi:10.3390/ani14060952)
Supplement: Supplementary file 1 [file animals-14-00952-s001.zip › animals-2896892-supplementary.pdf]

Supplementary Table S1. Introductory text and treatment profiles tested in quantitative survey (English-language version).

<table>
<tr><td colspan="2">
Please imagine the following situation:

• Your dog has short-term itch which will resolve itself within 14 days, but it should be treated in the meantime. *(**Alternative wording for chronic scenario**: Your veterinarian advises that your dog is now experiencing long-term itch, and it is uncertain when and if this condition will resolve itself.)*

• Your veterinarian offers you a choice of two treatment options.

• These are two different formulations of the same treatment. These both contain the same amount of the same medicine.

• Both formulations are equally effective and equally well tolerated but have different physical characteristics. *(**Alternative wording for chronic scenario:** Please assume that both formulations have been equally effective at treating short-term itch for your dog in the past. + The treatments have different physical characteristics and costs.)*

• On average, dogs have been observed to react differently to these formulations. Every dog is different, and the events described below are not guaranteed to occur. However, one formulation has been proven to be “palatable” in testing. “Palatable ” means that it is 80% likely that your dog will take this tablet voluntarily within five minutes of you offering the tablet.
</td></tr>
<tr><td><u>(Treatment A)</u></td><td><u>(Treatment B)</u></td></tr>
<tr><td>• An unflavored white tablet that can be given with or without food.
• You give this tablet twice daily.</td><td>• A chewable and pork liver-flavored “treat-like” chewable tablet that can be given with or without food (but there is typically no need to use food to administer the tablet).
• You give this tablet twice daily.</td></tr>
<tr><td>• On average, this treatment begins to take effect as quickly as 1 to 3 hours after administration, and is fully effective after 24 hours.</td><td>• On average, this treatment begins to take effect as quickly as 1 to 3 hours after administration, and is fully effective after 24 hours.</td></tr>
<tr><td>• You may have to disguise the tablet in a treat food that is suitable for them (or crumble the tablet into the food), for your dog to take the medication.
• This can take extra time, or cause trouble for you.
• Your dog may refuse to accept the tablet.</td><td>• Your dog typically accepts the chewable tablet as they would do a usual treat (from your hand or from an empty bowl), willingly accepting and eating the entire tablet.</td></tr>
</table>

• Your dog **may sometimes become distressed when you intervene** to ensure their tablet has been taken (for example, you may need to administer the tablet yourself by opening their mouth and putting the tablet on the back of the tongue, and confirming they have swallowed the tablet).<br>• You **may feel that this is harming your relationship** with your dog.	• You **typically do not need to intervene** to ensure your dog receives their tablet.<br>• Therefore, **your relationship with your dog is not changed significantly** beyond needing to give them this palatable tablet.
• If your dog refuses to accept their tablet, **you may be concerned that they will not experience the benefit** of the treatment. For example, you may worry that your dog has left their tablet somewhere in the house.<br>• You **may feel that this reflects badly** on your ability to provide appropriate care for your dog.	• As **your dog accepts the tablet, you are confident that they will experience the benefit** of the treatment.<br>• Therefore, **you feel more confident that you are successfully providing the medication** and **contributing to your pet's care**.
• **If your dog does not accept the tablet** in treat foods (or when crumbled into the food), **you will be concerned leaving your dog with other people** in case they are not able to administer the treatment.	• **You are not often concerned about leaving your dog with other people** as they are able to administer the tablet as if they are giving your dog a treat.
